# Supplementary material for: Microwave-Assisted Extraction Optimization and Effect of Drying Temperature on Catechins, Procyanidins and Theobromine in Cocoa Beans
Source: Molecules. 2023 Apr 27;28(9):3755. doi: 10.3390/molecules28093755 (PMC10180166; doi:10.3390/molecules28093755)
Supplement: Supplementary file 1 [file molecules-28-03755-s001.zip › molecules-2311355-supplementary.pdf]

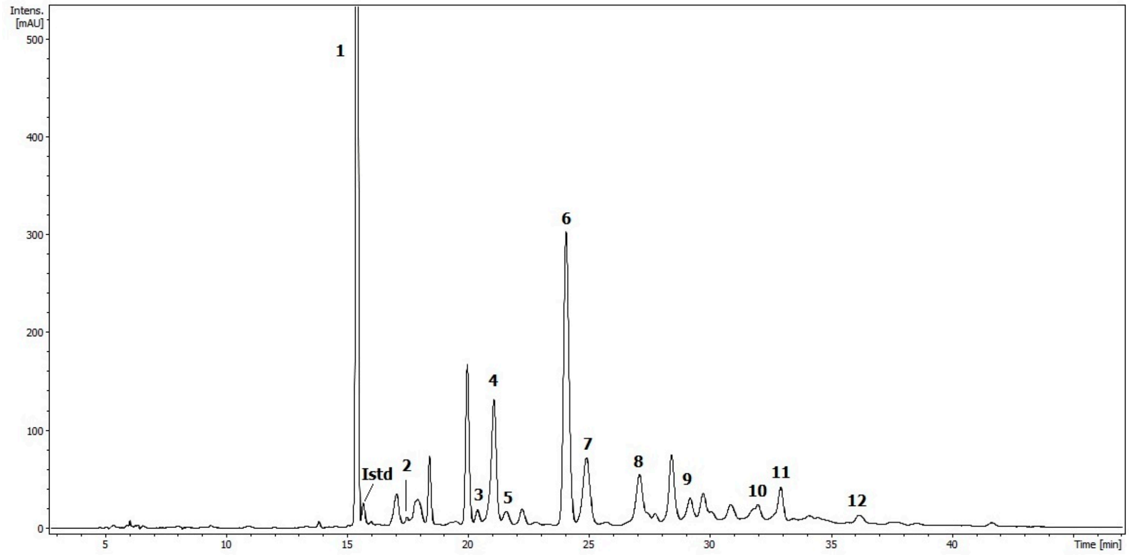

Figure S1. HPLC chromatogram (280 nm) of cocoa MAE extracts.

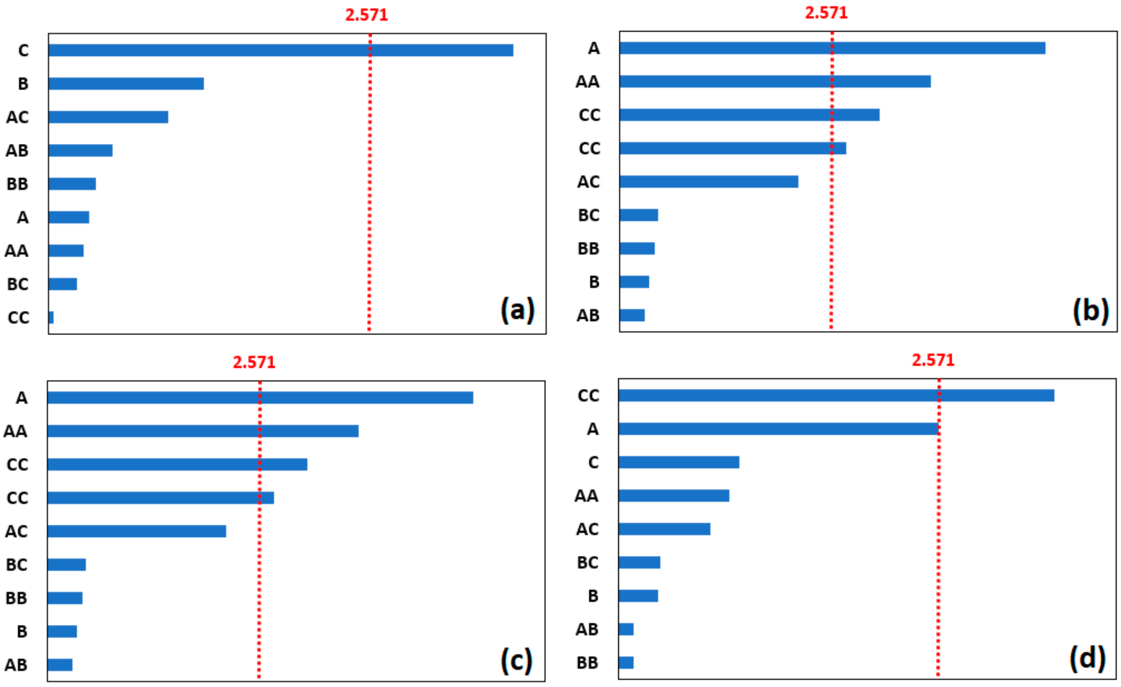

Figure S2. Pareto chart of standardized effects for the RSM model. (a) Dimers type A; (b) Dimers type B; (c) Trimer type B; (d) Tetramers type B. A: Temperature (°C), B: Time (min), C: Solvent composition (%MeOH). Bars exceeding the vertical line on the graph indicate that the corresponding factor terms are significant ($p < 0.05$).

Table S1. Regression equation in uncoded units for depend variables

		Equation
Dimer A	=	-18.1 + 0.135 T (°C) + 0.75 t (min) + 0.030 %MeOH - 0.00054 T (°C)*T (°C) - 0.0046 t (min)*t (min) - 0.00008 %MeOH*%MeOH - 0.00236 T (°C)*t (min) + 0.00177 T (°C)*%MeOH + 0.00107 t (min)*%MeOH
Dimer B	=	-3023 + 111.8 T (°C) + 9.4 t (min) + 93.3 %MeOH - 0.645 T (°C)*T (°C) - 0.46 t (min)*t (min) - 0.470 %MeOH*%MeOH + 0.127 T (°C)*t (min) - 0.356 T (°C)*%MeOH + 0.191 t (min)*%MeOH
Trimer B	=	-947 + 45.0 T (°C) + 7.6 t (min) + 62.2 %MeOH - 0.264 T (°C)*T (°C) - 0.220 t (min)*t (min) - 0.479 %MeOH*%MeOH + 0.023 T (°C)*t (min) - 0.147 T (°C)*%MeOH + 0.097 t (min)*%MeOH
Tetramer B	=	461 + 18.7 T (°C) - 13.3 t (min) + 57.3 %MeOH - 0.121 T (°C)*T (°C) + 0.102 t (min)*t (min) - 0.476 %MeOH*%MeOH - 0.041 T (°C)*t (min) - 0.097 T (°C)*%MeOH + 0.112 t (min)*%MeOH
